#  An Immune-Related lncRNA Expression Profile to Improve Prognosis Prediction for Lung Adenocarcinoma: From Bioinformatics to Clinical Word

**DOI:** 10.3389/fonc.2021.671341

**Published:** 2021-04-22

**Authors:** Boxiang Zhang, Rui Wang, Kai Li, Ziyang Peng, Dapeng Liu, Yunfeng Zhang, Liuzhi Zhou

**Affiliations:** ^1^ Department of Thoracic Surgery, The First Affiliated Hospital of Xi’an Jiaotong University, Xi’an, China; ^2^ Department of Surgery, The Second Affiliated Hospital, Zhejiang University School of Medicine, Hangzhou, China

**Keywords:** lung adenocarcinoma, immune-related lncRNA, clinical assessment, prognosis, biomarkers

## Abstract

**Background:**

Lung cancer is still the top-ranked cancer-related deaths all over the world. Now immunotherapy has emerged as a promising option for treating lung cancer. Recent evidence indicated that lncRNAs were also key regulators in immune system. We aimed to develop a novel prognostic signature based on the comprehensive analysis of immune-related lncRNAs to predict survival outcome of LUAD patients.

**Methods:**

The gene expression profiles of 491 LUAD patients were downloaded from TCGA. 1047 immune-related lncRNAs were obtained through Pearson correlation analysis of immune genes and lncRNAs using statistical software R language. Univariate and multivariate Cox regression analysis were performed to determine the optimal immune-related lncRNAs prognostic signature (ITGCB-DT, ABALON, TMPO-AS1 and VIM-AS1). Finally, we validated the immune-related lncRNAs prognostic signature in The First Affiliated Hospital of Xi’an Jiaotong University cancer center cohort.

**Results:**

A four immune-related lncRNAs prognostic signature was constructed to predict the survival outcome of LUAD patients. Statistical significance were found that the LUAD patients in high-risk group suffered shorter overall survival than those in low-risk group (P <0.001). ROC curve analysis shown that the four immune-related lncRNAs prognostic signature had the best predictive effect compared with age, gender, AJCC-stage, T stage, N stage, M stage (AUC = 0.756). More importantly, clinical cohort studies proved that the signature could predict the overall survival of LUAD patients with an AUC = 0.714.

**Conclusions:**

In summary, we demonstrated that the novel immune-related lncRNAs signature had the ability to predict the prognosis of LUAD patients, which might serve as potential prognostic biomarkers and guide the individualized treatment strategies for LUAD patients.

## Introduction

According to the latest global cancer statistics released in 2020, lung cancer is still the top-ranked cancer-related deaths all over the world, with a five-year survival rate of less than 19% (1, 2). In America, there were approximately 228,820 new cases of lung cancer and 135,720 deaths in 2020 (1). There are two major types of lung cancer, including small cell lung cancer (SCLC) and non-small cell lung cancer (NSCLC). NSCLC is further divided into three main subtypes, including lung squamous cell carcinoma (LUSC), lung adenocarcinoma (LUAD) and large cell lung cancer, of which LUAD accounts for about 40% (3). Since the signs of the early stage and clinical symptoms of LUAD are often non-specific and inconspicuous, a large proportion of patients are not diagnosed until the metastatic or advanced tumor stage (4). In recent years, although immunotherapy has shown unexpected anti-tumor effects in lung adenocarcinoma (5, 6). However, only a few patients have benefited from it, and there is no exact molecular stratification of the patients. This highlights the importance of investigation on new therapeutic approaches and novel biomarkers that provide prognostic information.

Long non-coding RNAs are a type of RNA molecule that are longer than 200 nucleotides and are not translated into proteins (7, 8). Once it was considered that these long non-coding RNAs were simply cloning artifacts or transcriptional noise with limited effects and single function in pre-RNA process (9). However, recent evidence indicated that lncRNAs were also involved in various biological functions in the cytoplasm and nucleus, such as transcriptional regulation, apoptosis, cell growth, tumorigenesis and epigenetic regulation (10, 11). It was well documented that lncRNAs played a comprehensive and complex regulatory role in cancer development and progression (12, 13). Moreover, lncRNAs have shown important regulatory effects of gene expression in immune system, including but not limited to immune activation, immune escape, immune surveillance, and immune infiltration (14, 15). For instance, the HCC-derived exosomal lncRNA TUC339 affected the complicated immune microenvironmental interaction between tumor and immune cells by regulating the polarization of M1/M2 macrophages (16). Besides, lncRNA GATA3-AS1 promoted tumor development and immune escape in triple negative breast cancer by destabilizing GATA3 but stabilizing PD-L1 (17). Thus, it is essential to develop an expression profile based on immune-related lncRNAs that can predict the prognosis of LUAD patients and further guide appropriate individualized treatment strategies.

In this study, we developed a novel prognostic signature based on the comprehensive analysis of immune-related lncRNAs in 491 adenocarcinoma patients downloaded from The Cancer Genome Atlas (TCGA). Four immune-related lncRNAs were confirmed to be related to immune response using univariate and multivariate Cox regression analysis. We then verified the expression of four immune-related lncRNAs on cells and tissues and further explored the relationship between prognostic signature and other clinicopathological characteristics. Finally, we confirmed that the novel immune-related lncRNAs signature had the ability to predict the prognosis of LUAD patients, which might serve as potential prognostic biomarkers and guide the individualized treatment strategies for LUAD patients.

## Materials and Methods

### Data Acquisition and Processing

The RNA-seq data of lung adenocarcinoma and matched normal tissues were downloaded from the GDC portal (https://portal.gdc.cancer.gov/) in December 2020, and the data was normalized using “limma” package. We further classified lncRNAs and protein-coding genes according to the gene annotations in Gencode (http://gencodegenes.org/). Immune genes were obtained from Molecular Signatures Database v7.1 using Gene Set Enrichment Analysis (MSigDB, https://www.gseamsigdb.org/gsea/index.jsp). Subsequently, we obtained immune-related lncRNA through Pearson correlation analysis of immune genes and lncRNAs using statistical software R language. Finally, the corresponding clinicopathological characteristics and survival information were acquired and integrated into RNA-seq data, including age, gender, stage, and TNM.

### Establishment of Prognostic Signature Using Immune-Related lncRNAs and Calculation of Risk Score

To determine the potential optimal immune-related prognostic lncRNAs, we performed univariate and multivariate Cox regression analysis on those immune-related lncRNA and survival data. Since the genes at the beginning of AC and AL belonged to conservative sequences and their functions had not been clearly clarified, we did not include them in the actual analysis. An HR value greater than one indicated an increased risk. Finally, four immune-related lncRNAs were identified and their regression correlation coefficients (β) with the lowest AIC values. We then established the optimal immune-related lncRNAs prognostic signature and calculated the risk scores of each lung adenocarcinoma patient based on the expression levels of immune-related lncRNAs and the Risk coefficients (β):Risk scores =0.411143 × Expression_ABALON_ − 0.259290 × Expression_VIM-AS1_ + 0.337683 × Expression_TMPO-AS1_ + 0.265425 × Expression_ITGB1-DT_.

### Cell Culture and Real-Time Quantitative PCR

To further confirm the expressions of the four immune-related lncRNAs in cells and tissues, human normal lung epithelial cell (B2B) and lung adenocarcinoma cell lines (A549, H1299, H1975) were obtained from the laboratory and cultured in DMEM medium containing 10% FBS with 1% penicillin and streptomycin in a humidified incubator. The total RNA of various cell lines was extracted using RNA-Quick purification kit according to manufacturer’s instructions (Vazyme, RN001). Total RNA were reversed into cDNA using PrimeScript™ RT reagent kit with gDNA Eraser (Takara, RR047A). Next, we performed quantitative PCR to determine the relative expression levels of the four lncRNAs (Takara, RB820A). The tissues’ expressions and survival curves of the four lncRNAs were acquired from the GEPIA database (http://gepia.cancer-pku.cn/). The expression of β-actin was used as an endogenous control. All samples were analyzed using comparative 2^−ΔΔC^ method. All primers’ sequences used in PCR were shown in **Table S1**.

### Predictive Analysis of Immune-Related lncRNAs Risk Score Signature

All LUAD patients were divided into high and low risk groups according to the median of risk score as threshold. We compared the survival curves of the two groups using the Kaplan–Meier method with log-rank test. ROC curve and AUC value were used to assess the accuracy of the immune-related signature. Further, univariate and multivariate Cox regression analysis were utilized to evaluate clinicopathological characteristics related to prognosis. The heat map shows the differences of the four lncRNAs in two groups. Finally, to explore the influence of single lncRNA on LUAD patients in our prognostic model, we explored the relationship of single lncRNA and clinicopathological characteristics with student’s t-test.

### Principal Components Analysis and Immune Infiltration

Principal Components Analysis was utilized to visualize the prognostic model. Immune Response and Immune System Process sets were acquired from MSigDB for subsequent analysis. Gene Set Enrichment Analysis were performed on the DEGs in high- and low-risk groups with P <0.05 and |log (fold change) >1|. Cibersort was conducted to evaluate the immune infiltrating cells in each sample with the Pfilter <0.05.

### Validation of Immune-Related lncRNAs Prognostic Signature in The First Affiliated Hospital of Xi’an Jiaotong University Cancer Center Cohort

To further screen and verify the prognostic signature, we collected lung adenocarcinoma and adjacent tissues of 78 LUAD patients from the First Affiliated Hospital of Xi’an Jiaotong University, who underwent surgical resection from January 2011 to December 2013. All included patients were diagnosed with LUAD by histopathological examination and did not receive any radiotherapy, chemotherapy, or immunotherapy. We examined the four immune-related lncRNAs and compared them with other clinicopathological characteristics. All patients enrolled were written informed consent. The study was supported by the Ethics Committee of the Affiliated Hospital of Xi’an Jiaotong University.

### Statistical Analysis

All computations were conducted using R software (version 4.0.4). Associations between risk scores and other clinicopathological features in LUAD patients were analyzed with Fisher exact test or chi-square test. Kaplan–Meier method and log-rank analysis were performed to assess survival data. Univariate and multivariate Cox regression analyses were performed to assess independent prognostic factors. P <0.05 was considered statistically significant.

## Results

### Construction and Assessment of Immune-Related lncRNAs Signature

**Figure 1:**
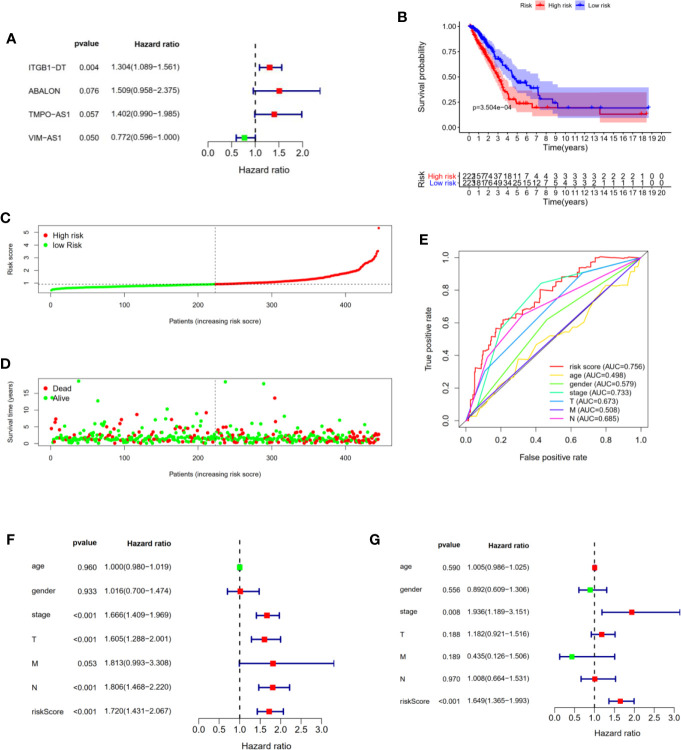
Construction and evaluation of four immune-related lncRNAs prognostic signature for LUAD patients. **(A)** The forest plot shown the P values, Hazard Ratios of four immune-related lncRNAs from multivariate Cox regression analysis. **(B)** Kaplan–Meier analysis illustrated that high risk group had poor prognosis and shorter overall survival in LUAD patients. **(C)** Risk scores of LUAD patients were sorted with the signature. **(D)** The scatter plot of risk scores and survival status in LUAD patients. **(E)** ROC curve analysis demonstrated that risk scores (AUC = 0.756) had the highest reliability and accuracy compared with age, gender, stage, TNM. **(F, G)** Univariate and multivariate Cox regression analysis of prognostic factors combined with other clinicopathological features.

A total of 497 LUAD samples and 54 matched normal controls were available from TCGA database. Subsequently, we downloaded 131 lncRNAs as well as their expression profiles and screened out 331 immune genes from TCGA. Then, 1047 immune-related lncRNAs were obtained using Person correlation analysis with the standard P <0.05 and |R| >0.8. Finally, univariate and multivariate Cox regression analysis were performed to further filter out potential prognostic lncRNAs from those immune-related lncRNAs, and four immune-related lncRNAs were found to be significantly associated with the LUAD patients overall survival (**Figure 1A** and **Table 1**).

**Table 1:** The HRs, P-value, and Coef of four immune-related lncRNAs in the multivariate Cox regression analysis.

LncRNAs	HR (95% CI)	P-value	Coef
ITGB1-DT	1.3040 (1.0894–1.5608)	0.004	0.265
ABALON	1.509 (0.958–2.375)	0.076	0.411
TMPO-AS1	1.402 (0.990–1.985)	0.057	0.337
VIM-AS1	0.772 (0.596–1.000)	0.050	−0.212

HR, hazard ratio; Coef, regression coefficient; CI, confidence interval.

Next, we constructed a four immune-related lncRNAs prognostic signature to predict the survival outcomes of LUAD patients. We calculated the risk score of each patient using the following scheme: Risk scores = 0.411143 × Expression_ABALON_ − 0.259290 × Expression_VIM-AS1_ + 0.337683 × Expression_TMPO-AS1_ + 0.265425 × Expression_ITGB1-DT_. Furthermore, all LUAD patients from the TCGA data sets were divided into high and low risk groups according to the median of risk scores as threshold. We found that the LUAD patients in high risk group suffered shorter overall survival than those in low-risk group with statistical significance (**Figure 1B**). Subsequently, we sorted the risk scores of all LUAD patients and then evaluated their survival status distribution based on the four immune-related prognostic risk scores. The survival status analysis indicated that the LUAD patients had shorter overall survival and higher mortality with the risk scores increasing (**Figures 1C, D**). Next, we assessed the prediction accuracy of the prognostic signature based on the four immune-related lncRNAs through time-independent ROC curve analysis. The ROC curve analysis shown that the four immune-related lncRNAs’ prognostic signature had the best predictive effect compared with age, gender, AJCC-stage, T stage, N stage, M stage (AUC = 0.756) (**Figure 1E**). These results demonstrated that our four immune-related lncRNAs’ prognostic signature was capable of predicting the survival outcomes of LUAD patients.

In addition, to prove that the four immune-related lncRNAs prognostic model we constructed could be used as independent prognostic predictions for LUAD patients, we performed univariate and multivariate Cox regression analysis with the following clinicopathological characteristics: age, gender, AJCC-stage, T stage, N stage, M stage. Univariate Cox regression analysis shown that AJCC-stage (P <0.001), T stage (P <0.001), N stage (P <0.001), risk score (P <0.001) were associated with the prognostic survival in LUAD patients (**Figure 1F**). Multivariate Cox regression analysis indicated that AJCC-stage (P = 0.008), risk score (P <0.001) were still significantly associated with overall survival, and four immune-related lncRNAs prognostic signature could be identified as an independent prognostic factor in LUAD patients (**Figure 1G**).

### Evaluating Immune-Related lncRNAs Expressions in Cells and Tissues

**Figure 2:**
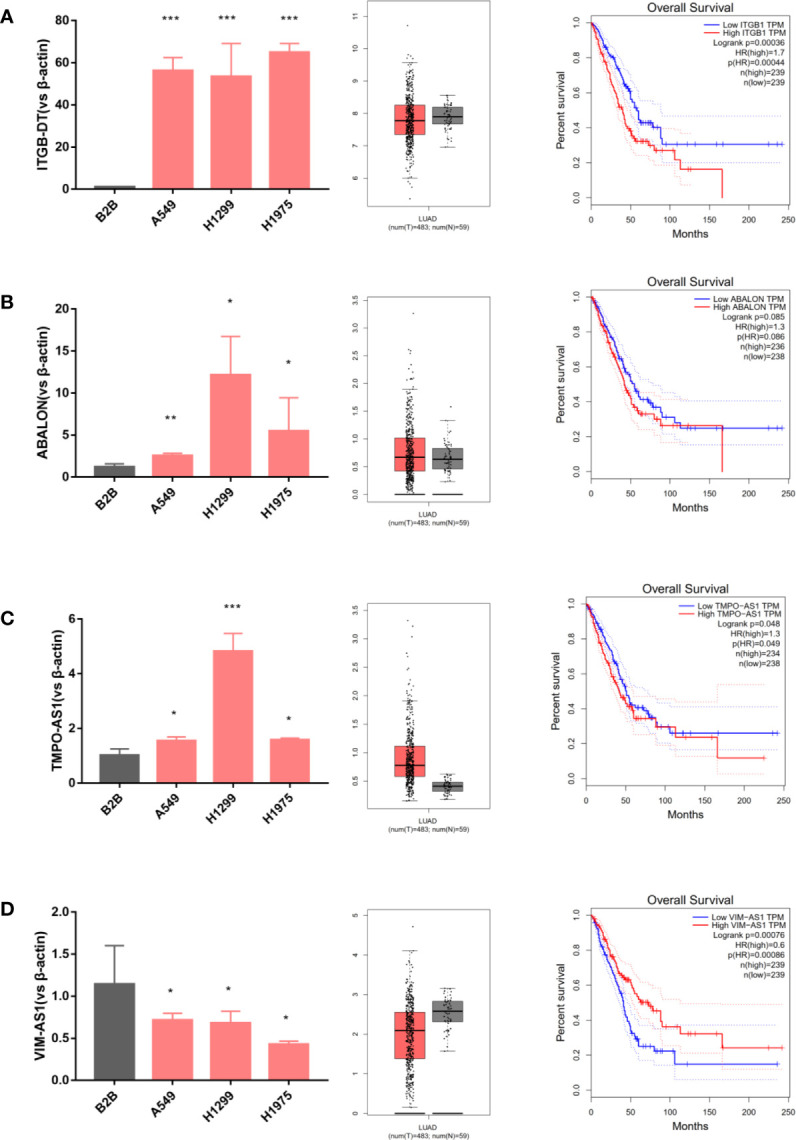
Evaluating immune-related lncRNAs expression in cells and tissues. ITGCB-DT, ABALON and TMPO-AS1 expressions were relatively highly expressed in LUAD cell lines and LUAD tissues compared with normal controls and LUAD patients had worse prognosis with their increased expressions. The opposite of VIM-AS1. **(A)** ITGB-DT **(B)** ABALON **(C)** TMPO-AS1 **(D)** VIM-AS1. *P < 0.05; **P < 0.01; ***P < 0.001.

To further identify the four immune-related lncRNAs expression profiles, we analyzed their relative expression levels in LUAD cell lines (A549, H1299, H1975) and normal lung cell (B2B) using quantitative PCR. Next, the Gepia database was used to obtain the expressions of four immune-related lncRNAs in LUAD tissues and adjacent normal tissues. Moreover, we further downloaded the survival curves of four immune-related lncRNAs from the Gepia database. As shown in **Figures 2A–C**, the ITGCB-DT, ABALON and TMPO-AS1 expressions were relatively highly expressed in LUAD cell lines (A549, H1299, H1975) compared with normal control (B2B). The same results were also found in clinical samples that ITGCB-DT, ABALON as well as TMPO-AS1 expressions were significantly up-regulated in LUAD tumor tissues compared with adjacent tissues and LUAD patients with higher expressions of ITGCB-DT, ABALON and TMPO-AS1 tended to have shorter overall survival and worse prognosis. In contrast, VIM-AS1 was relatively lower expressed in A549, H1299 and H1975 as well as LUAD tissues, and LUAD patients with higher expression of VIM-AS1 had better prognosis and longer overall survival (**Figure 2D**). Thus, these results indicated that ITGCB-DT, ABALON, TMPO-AS1 and VIM-AS1 could serve as independent prognostic biomarkers in LUAD.

### Correlations With Clinicopathological Characteristics

**Figure 3:**
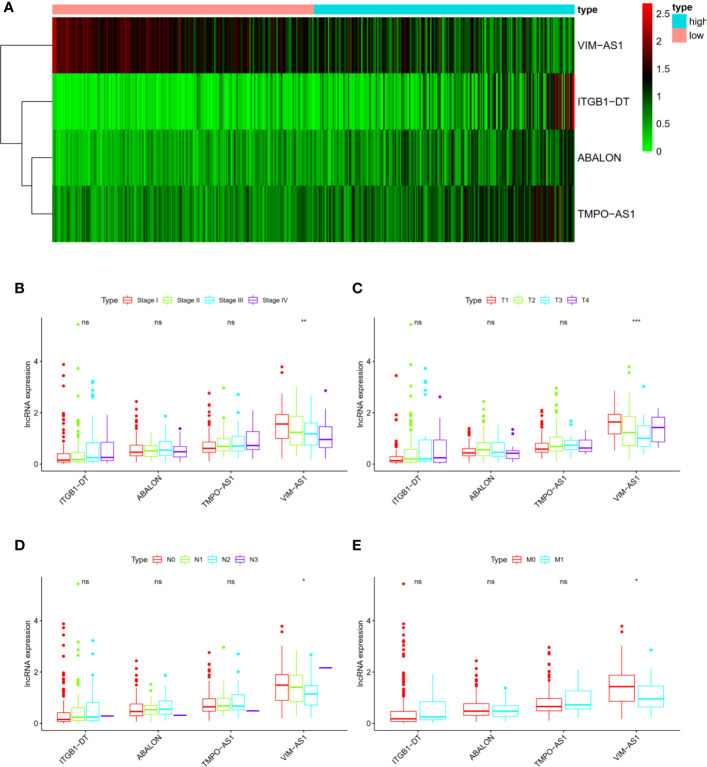
The associations between four immune-related lncRNAs and other clinicopathological characteristics. **(A)** Heat map shown the expression levels of four immune-related lncRNAs in high- and low- risk groups. **(B–E)** The relationships of four immune-related lncRNAs and AJCC stage as well as AJCC TNM. *P < 0.05; **P < 0.01; ***P < 0.001; ns, no significance.

We also analyzed the associations between four immune-related lncRNAs and clinicopathological characteristics to explore the impact of single lncRNA in LUAD patients. The heat map shown that the four immune-related lncRNAs were obviously differentially expressed in high- and low- risk patients, of which ITGB1-DT, ABALON as well as TMPO-AS1 was up-regulated, and VIM-AS1 was down-regulated in high risk group (**Figure 3A**). In terms of single lncRNA, no statistically difference was found in the expression levels of ITGB1-DT, ABALON as well as TMPO-AS1 with AJCC-stage (**Figure 3B**), T stage (**Figure 3C**), N stage (**Figure 3D**) and M stage (**Figure 3E**). However, it could be found that there was a trend that ITGB1-DT, ABALON and TMPO-AS1 were increased with AJCC-stage and TNM stage. Besides, the expression level of VIM-AS1 was negatively associated with AJCC-stage (**Figure 3B**), T stage (**Figure 3C**), N stage (**Figure 3D**) and M stage (**Figure 3E**). These results were basically consistent with our above analysis, proving that our four-immune related lncRNAs prognostic signature was competent for predicting survival prognosis in LUAD patients.

**Figure 4:**
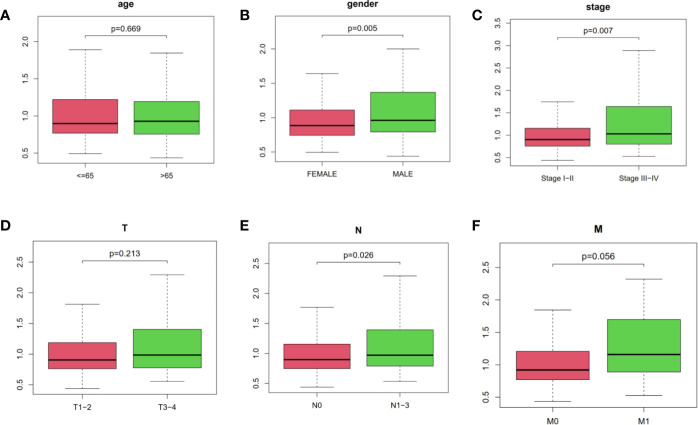
The relationships of risk score and age, gender, AJCC stage as well as AJCC-TNM. **(A)** age, **(B)** gender, **(C)** stage, **(D–F)** AJCC-TNM, respectively. The ordinate was risk score.

In addition, we performed chi-square test to explore the associations of risk score and other clinicopathological characteristics in LUAD patients. As shown in **Figure 4**, there were significant differences between high- and low-risk groups in gender (P = 0.005, **Figure 4B**), AJCC stage (P = 0.007, **Figure 4C**), N stage (P = 0.026, **Figure 4E**), and M stage (P = 0.056, **Figure 4F**). These results proved that our four immune-related lncRNAs profiles could play a potential role in predicting tumor progression and survival prognosis of LUAD patients.

### Functional and Pathway Enrichment Analysis of DEGs Related to Risk Score

**Figure 5:**
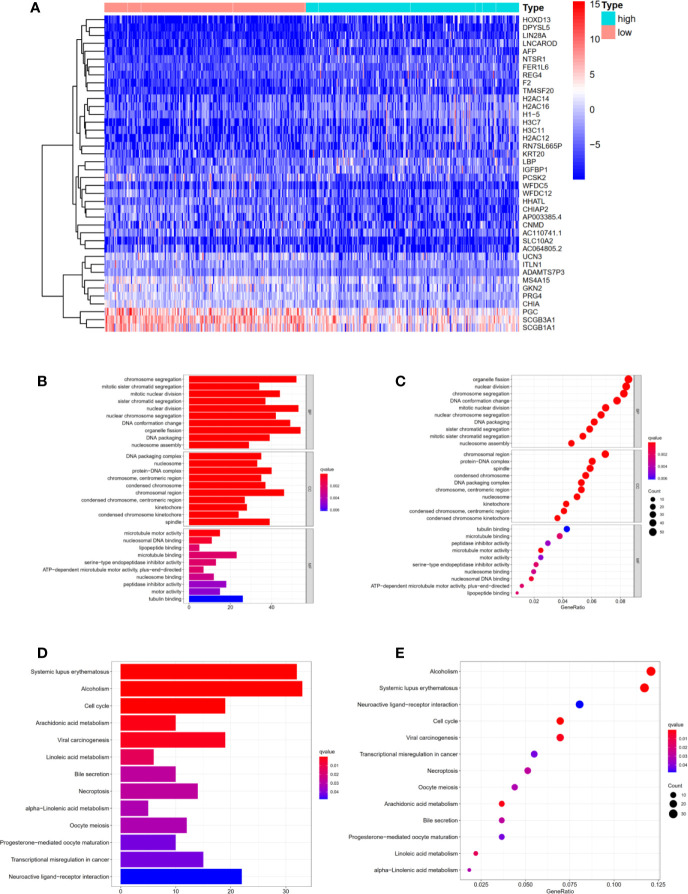
Functional and Pathway enrichment analysis of DEGs related to risk score. **(A)** The heat map displayed DEGs in high- and low-risk groups **(B, C)** Functional enrichment analysis of DEGs using Gene Ontology. **(D, E)** Pathway enrichment analysis of DEGs using KEGG method. KEGG, Kyoto Encyclopedia of Genes and Genomes.

Differentially expressed genes in high- and low- risk groups were analyzed with R software. 1029 DEGs were available using “Limma” package with the criteria P <0.05 and log (fold change) >1 (**Figure 5A**). Next, we performed Gene Ontology and KEGG analysis to further explore the DEGs’ function and pathway enrichment. Obviously, in Biological Process, Cellular Component and Molecular Function, the differentially expressed genes were mainly enriched in organelle fission, chromosomal region, tubulin binding (**Figures 5B, C**). In addition, KEGG analysis shown that these risk-related DEGs were significantly enriched in alcoholism, systemic lupus erythematosus, neuroactive ligand–receptor interaction (**Figures 5D, E**). Moreover, we performed Gene Set Enrichment Analysis (Immunologic signatures) for the high-risk group. The top 10 differentially enriched immunologic signatures were shown in **Table S2**. We explained the risk-related DEGs in LUAD patients from a mathematical perspective, which might promote future research and treatment of LUAD.

### Identification of Immune Status in High- and Low-Risk Groups

**Figure 6:**
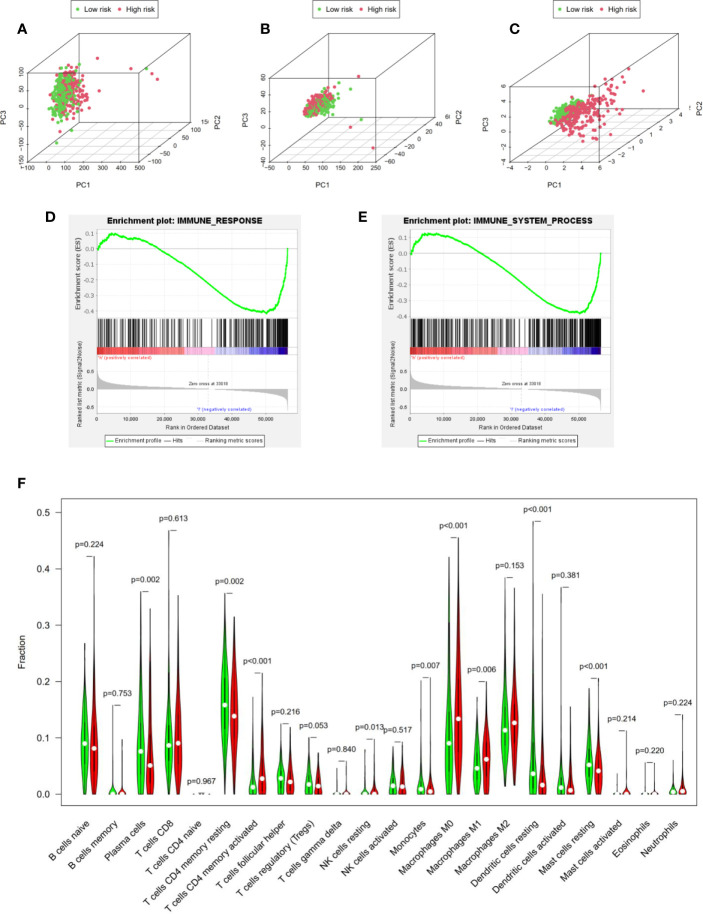
Identification of immune status in high- and low-risk groups. **(A**–**C)** Principal component analysis demonstrated that significant differences were found in the immune status between two risk groups according to four immune-related lncRNAs. Whole gene expression profiles, all immune-related lncRNAs, four risk genes, respectively. GSEA analysis shown that both immune response **(D)** and immune system process **(E)** were enriched in the low risk group. **(F)** Cibersort algorithm calculated the difference of immune infiltration in high- and low-risk groups.

Based on principal component analysis, we further analyzed the distinct distribution of low- and high-risk groups using whole gene expression profiles, all immune-related lncRNAs and four risk genes. The results shown that the samples were not significantly separated into two sections and the immune status of LUAD patients were overlapped between high- and low- risk groups based on whole gene expression profiles and all immune-related lncRNAs sets (**Figures 6A, B**). However, significant differences were found in the immune status of high risk groups compared with low risk groups according to the four risk genes sets (**Figure 6C**).

Besides, GSEA analysis was performed and the results indicated that both immune response (**Figure 6D**) and immune system process (**Figure 6E**) were enriched in the low risk group. In addition, to further explore immune infiltration in high- and low-risk groups, we analyzed 22 immune infiltrating cells in the LUAD microenvironment based on the Cibersort algorithm. We calculated the 22 kinds of immune infiltrating cells with Pfilter <0.05 in each LUAD sample. Moreover, “Vioplot” and “limma” packages were used to visualize the immune infiltrating cells in high- and low- risk groups. As shown in **Figure 6F**, plasma cells (P = 0.002), memory CD4+ T cells (P <0.001), NK cells (P = 0.013), monocytes (P = 0.007), M1 macrophages (P = 0.006), dendritic cells (P <0.001) and mast cells (P <0.001) demonstrated significant differences, indicating that our four immune-related lncRNAs signature could distinguish immune infiltration in LUAD patients. In a word, our four immune-related lncRNAs prognostic signature was component to distinguish the immune status and predict the survival prognosis of LUAD patients.

### Identification and Validation of Prognostic Signature in Clinical Cancer Cohort

**Figure 7:**
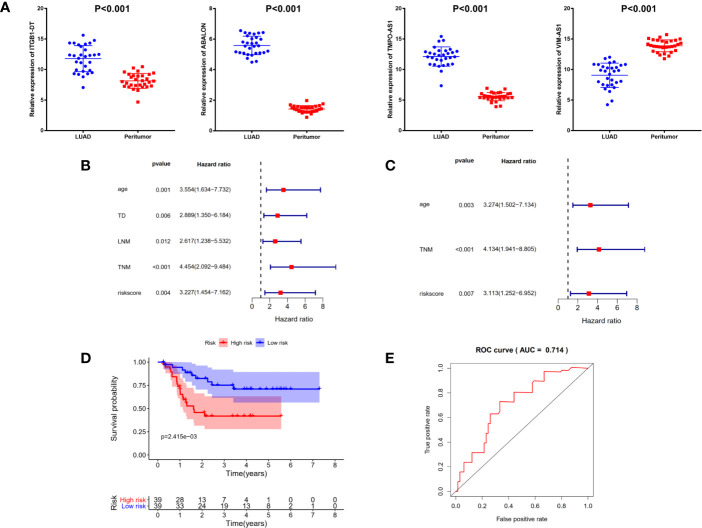
Identification and validation of prognostic signature in clinical cancer cohort. **(A)** The expressions of ITGB1-DT (P <0.001), ABALON (P <0.001) as well as TMPO-AS1(P <0.001) were significantly up-regulated in LUAD tissues compared with adjacent LUAD tissue, while VIM-AS1 (P <0.001) expressed the opposite. **(B)** Univariate Cox regression analysis demonstrated that age, tumor diameter, lymph node metastasis, TNM stage and risk scores were related to the survival prognosis of LUAD patients **(C)** Multivariate Cox regression analysis shown that age, TNM stage and risk score could serve as independent prognosis biomarkers in LUAD patients. **(D)** Kaplan–Meier analysis confirmed four immune-related lncRNAs signature could predict the prognosis of LUAD patients in actual clinical cohort. **(E)** ROC curve analysis shown that the signature was reliable and accurate (AUC = 0.714).

To further validate the signature we constructed, we analyzed the expressions of four immune-related lncRNAs in 78 LUAD samples and 30 matched normal controls from clinical cancer cohort. As shown in **Figure 7A**, the expressions of ITGB1-DT (P <0.001), ABALON (P <0.001) as well as TMPO-AS1 (P <0.001) were significantly up-regulated in LUAD tissues compared with adjacent LUAD tissue, while VIM-AS1 (P <0.001) expressed the opposite, which were consistent with our previous analysis from TCGA database.

Additionally, to explore the relationships between risk score and other clinicopathological characteristics, we calculated the risk scores of 78 LUAD patients according to the four immune-related lncRNAs signature we established before. All 78 LUAD patients were divided into high- and low-risk subgroups with the median of risk score as threshold. Chi-square test analysis demonstrated that high risk scores were related with weight-loss (P = 0.009), tumor diameter (P <0.001), multiple lesions (P <0.001), lymph node metastasis (P = 0.011), differentiation (P <0.001) and TNM stage (P <0.001) (**Table 2**). However, there was no significant difference between risk score and other clinicopathological characteristics, such as age, smoking and vessel carcinoma embolus.

**Table 2:** Associations between risk scores and clinicopathological characteristics in clinical LUAD cohort.

Characteristics	Total	Risk Scores		*P* value
		Low (n = 39) 1.69	High (n = 39) 1.62	
Age, ys				0.172
≤65	43	25	18	
>65	35	14	21	
Gender				0.651
Female	44	17	20	
Male	34	22	19	
Smoking				0.084
Absent	54	31	23	
Present	24	8	16	
Weight-loss				0.009*
≤5%	30	21	9	
>5%	48	18	30	
Tumor diameter				<0.001*
≤5 cm	58	37	21	
>5 cm	20	2	18	
Multiple lesions				<0.001*
Absent	36	27	9	
Present	42	12	30	
Vessel carcinoma embolus				0.999
Absent	74	37	37	
Present	4	2	2	
Lymph node metastasis				0.011*
Absent	46	29	17	
Present	32	10	22	
Atelectasis				0.494
Absent	76	37	39	
Present	2	2	0	
Differentiation				<0.001*
Low	46	14	32	
High/Moderate	32	25	7	
TNM stage				<0.001*
I–II	51	34	17	
III–IV	25	4	21	

TNM, tumor-node-metastasis; *P <0.05 was considered statistically significant; Values are mean ± standard deviation or n (%).

Next, to confirm the independent prognostic factors in LUAD patients, we performed univariate and multivariate Cox regression analysis on various clinicopathological characteristics and risk scores. Univariate Cox regression analysis demonstrated that age, tumor diameter, lymph node metastasis, TNM stage and risk scores were related to the survival prognosis of LUAD patients (**Figure 7B** and **Table 3**). Multivariate Cox regression analysis shown that age, TNM stage and risk score could serve as independent prognosis biomarkers in LUAD patients (**Figure 7C** and **Table 3**). Finally, a significant statistical difference was found in overall survival of high- and low-risk groups (P <0.001) (**Figure 7D**). And through ROC curve analysis, we demonstrated that the four immune-related lncRNAs signature has high accuracy and reliability in predicting the prognosis of LUAD patients in actual clinical application (AUC = 0.714, **Figure 7E**).

**Table 3:** Prognosis analysis of overall survival in clinical LUAD cohort.

Clinicopathologic parameters	Univariate analysis		Multivariate analysis	
	HR (95%CI)	*P*	HR (95%CI)	*P*
Age (≤65 vs >65)	3.554 (1.634–7.732)	0.001*	3.225 (1.432–7.263)	0.005*
Gender (Female vs Male)	0.901 (0.433–1.874)	0.779		
Smoking (Absent vs Present)	1.191 (0.542–2.620)	0.663		
Weight-loss (≤5% vs >5%)	1.884 (0.805–4.412)	0.145		
Tumor diameter (≤5 cm vs >5 cm)	2.889 (1.350–6.184)	0.006*	1.282 (0.490–3.355)	0.612
Multiple lesions (Absent vs Present)	1.624 (0.766–3.445)	0.206		
Vessel carcinoma embolus (Absent vs Present)	1.018 (0.242–4.287)	0.981		
Lymph node metastasis (Absent vs Present)	2.617 (1.238–5.532)	0.012*	1.002 (0.375–2.680)	0.997
Atelectasis (Absent vs Present)	1.665 (0.224–12.399)	0.619		
Differentiation (Low vs High/Moderate)	0.510 (0.231–1.124)	0.095		
TNM stage	4.454 (2.092–9.484)	<0.001*	3.266 (1.236–8.631)	0.017*
Risk score	3.227 (1.454–7.162)	0.004*	3.113 (1.252–6.952)	0.007*

LUAD, lung adenocarcinoma; TNM, tumor-node-metastasis; HR, hazard ratio; *P <0.05 was considered statistically significant.

## Discussion

Although surgical resection had been proven to be essential for early lung adenocarcinoma and made great progress in the past thirty years, the treatment for advanced and metastatic lung adenocarcinoma was still unsatisfactory (18). For some LUAD patients with similar clinical symptoms, there were significant differences in survival outcomes due to genetic heterogeneity. Therefore, in addition to traditional clinical risk indicators, exploring novel prognostic molecular classification for LUAD was crucial. With the development of bioinformatics analysis and third-generation sequencing technology, it had been confirmed that lncRNAs were involved in tumorigenesis and cancer development (19, 20). Increased studies had shown the importance of lncRNAs in LUAD, such as carcinogenic functions (21), tumor suppressor (22) and prognosis biomarkers (23). Recently, lncRNAs were emerging as key regulators in immune system. Therefore, it was urgent to explore the immune-related lncRNAs in LUAD and its relationship with immune cell infiltration.

In this study, 1,047 immune-related lncRNAs were obtained from MSigDB using Person correlation method for further subsequent analysis. The main finding of our research was that we constructed a four immune-related lncRNAs prognostic signature and verified its stability and reliability through ROC curve and real world data. We demonstrated that our prognostic signature was significantly related with OS and could distinguish LUAD patients with good or poor prognosis based on the four lncRNAs. Our signature had been proven to be an independent prognostic factor in LUAD patients through univariate and multivariate Cox regression analysis. Different from the past immune-related lncRNA prognostic studies, we removed the conservative sequence genes at the beginning of AC or AL when constructing the model, further tested and verified our immune-related lncRNAs on cells and clinical samples. In addition, we also investigated the relationships of single lncRNA expression and clinicopathological characteristics. The results show that ABALON, ITGB-DT and TMPO-AS1 were risk-related genes, while VIM-AS1 was regarded as risk protective genes. PCA analysis indicated that our signature could clearly distinguish high- and low-risk groups compared with whole gene expression profiles or all immune-related lncRNAs. Finally, we validated the four immune-related lncRNAs prognostic model on clinical LUAD patients cohort. These findings proved that the four immune-related lncRNAs prognosis signature was related to the survival prognosis of LUAD patients, and could potentially guide clinicians in the treatment of LUAD patients.

As we know, lncRNAs participated in tumor development (including LUAD) through various mechanisms. Previous studies have indicated that up-regulation of lncRNA UCA1, TTN-AS1 and FEZF1-AS1 in LUAD were related to poor prognosis, and down-regulation of LCAL62 also promoted tumor progression and invasion. In addition, Pan et al. indicated that lncRNA JPX regulated tumorigenesis and metastasis of lung cancer through JPX/miR-33a-5p/Twist1 axis and activating Wnt/beta-catenin signaling (24). Peng et al. show that LINC00312 induced LUAD migration and vasculogenic mimicry through directly binding to transcription factor Y-Box Binding Protein 1 (YBX1) (25). As previously reported, LncRNA HMMR-AS1 was significantly upregulated in LUAD and promoted proliferation and metastasis of lung adenocarcinoma by regulating MiR-138/sirt6 axis (26). On the other hand, Mu et al. reported that lncRNA TMPO-AS1 promoted lung adenocarcinoma progression and was negatively regulated by miR-383-5P (27). Despite great progress have achieved in the lncRNA research, the function and molecular mechanism of most lncRNA were still unclear and need further investigation.

In recent years, given that immunotherapy become the dawn of cancer treatment, it has been a hotspot to construct immune-related lncRNA signature to predict tumor prognosis. For instance, Shen et al. constructed a 11-lncRNA prognostic signature for breast cancer, which was associated with immune infiltrating cell subtypes (28). Zhou et al. reported an immune-related six-lncRNA signature to improve prognosis prediction for glioblastoma multiforme (29). In this study, we found that high expression of ITGB1-DT, ABALON as well as TMPO-AS1 and low expression of VIM-AS1 in lung adenocarcinoma were associated with poor prognosis. We successfully constructed four immune-related lncRNA prognostic signature and validated them in clinical cancer cohort for the first time.

In conclusion, we identified a four immune-related lncRNAs signature that had the ability to predict the prognosis of LUAD patients and was validated by clinical cancer cohort, which might serve as potential prognostic biomarkers and guide the individualized treatment strategies for LUAD patients.

## Data Availability Statement

The datasets presented in this study can be found in online repositories. The names of the repository/repositories and accession number(s) can be found in the article/**Supplementary Material**.

## Ethics Statement

The studies involving human participants were reviewed and approved by the Ethics Committee of the Affiliated Hospital of Xi’an Jiaotong University. All patients enrolled were written informed consent. The patients/participants provided their written informed consent to participate in this study.

## Author Contributions

LZ and BZ conceived and designed the study. BZ, RW, KL, and ZP conducted the study. DL contributed to the acquisition of data. BZ and RW analyzed the data. LZ, ZB and RW interpreted the data. LZ, BZ, YZ, and RW reviewed and edited the manuscript. All authors contributed to the article and approved the submitted version.

## Funding

This work was supported by Key Research and Development Program of Shaanxi (Program No.2019SF-044 & No.2019SF-129).

## Conflict of Interest

The authors declare that the research was conducted in the absence of any commercial or financial relationships that could be construed as a potential conflict of interest.

## Abbreviations

LUAD, lung adenocarcinoma; OS, overall survival; AUC, area under the curve; TD, tumor diameter; LNM, lymph node metastasis; TCGA, The Cancer Genome Atlas; GSEA, Gene set enrichment analysis; TNM, tumor-node-metastasis; HR, hazard ratio.
