# Supplementary material for: An Immune-Related lncRNA Expression Profile to Improve Prognosis Prediction for Lung Adenocarcinoma: From Bioinformatics to Clinical Word
Source: Front Oncol. 2021 Apr 22;11:671341. doi: 10.3389/fonc.2021.671341 (PMC8100529; doi:10.3389/fonc.2021.671341)
Supplement: Supplementary file 1 [file Table_1.docx]

**Table S1. Primers’ sequences**

| Genes’ names | Primers | Sequences (5’ → 3’) |
| --- | --- | --- |
| ITGB1-DT | Forward | GAACATGCCTCCACCATACCACTG |
|  | Reverse | ACTTTGAGAAGCTGCCTCTTTGGG |
| ABALON | Forward | CCCGTCTTCTCCGAAATGCCTTC |
|  | Reverse | CACCTGCCTGCCTTTGCCTAAG |
| TMPO-AS1 | Forward | GGACACAAAGCCAAGCCAGACC |
|  | Reverse | TGCTCGCCTCCTGCCTGTAG |
| VIM-AS1 | Forward | GAATGGGCACTGGTCCTCTTCATC |
|  | Reverse | ACTGTGGTATTTGCTGCTGTTCCC |
| β-actin | Forward | ATCGTGCGTGACATTAAGGAGAAG |
|  | Reverse | AGGAAGGAAGGCTGGAAGAGTG |
