# Supplementary material for: An Immune-Related lncRNA Expression Profile to Improve Prognosis Prediction for Lung Adenocarcinoma: From Bioinformatics to Clinical Word
Source: Front Oncol. 2021 Apr 22;11:671341. doi: 10.3389/fonc.2021.671341 (PMC8100529; doi:10.3389/fonc.2021.671341)
Supplement: Supplementary file 2 [file Table_2.docx]

**Table S2: The top 10 differentially enriched immunologic signatures in high risk group**

| **Description** | **size** | **ES** | **NES** | **p** | **q** | **Rank at max** |
| --- | --- | --- | --- | --- | --- | --- |
| GSE7460_CTRL_VS_TGFB_TREATED_ACT_FOXP3_HET_TCONV_UP | 200 | 0.66 | 2.67 | 0.000 | 0.000 | 6679 |
| GSE45365_HEALTHY_VS_MCMV_INFECTION_CD11B_DC_IFNAR_KO_DN | 191 | 0.63 | 2.64 | 0.000 | 0.000 | 5319 |
| GSE7460_CTRL_VS_TGFB_TREATED_ACT_TREG_UP | 200 | 0.71 | 2.64 | 0.000 | 0.000 | 4144 |
| GSE40666_WT_VS_STAT1_KO_CD8_TCELL_WITH_IFNA_STIM_90MIN_DN | 200 | 0.73 | 2.63 | 0.000 | 0.000 | 3698 |
| GSE8685_IL2_STARVED_VS_IL2_ACT_IL2_STARVED_CD4_TCELL_DN | 197 | 0.68 | 2.61 | 0.000 | 0.000 | 5078 |
| GSE21063_WT_VS_NFATC1_KO_BCELL_DN | 200 | 0.69 | 2.61 | 0.000 | 0.000 | 5849 |
| GSE45365_WT_VS_IFNAR_KO_CD11B_DC_MCMV_INFECTION_DN | 189 | 0.65 | 2.61 | 0.000 | 0.000 | 5041 |
| GSE10239_NAIVE_VS_KLRG1HIGH_EFF_CD8_TCELL_DN | 199 | 0.74 | 2.61 | 0.000 | 0.000 | 5240 |
| GSE10239_NAIVE_VS_DAY4.5_EFF_CD8_TCELL_DN | 200 | 0.80 | 2.61 | 0.000 | 0.000 | 3392 |
| GSE39110_UNTREATED_VS_IL2_TREATED_CD8_TCELL_DAY3_POST_IMMUNIZATION_DN | 200 | 0.69 | 2.61 | 0.000 | 0.000 | 4495 |
